# Age Inflection Points of Colorectal Adenoma Risk in Young Adults: A Joinpoint Regression Analysis of a Single-Center Retrospective Colonoscopy-Based Cohort

**DOI:** 10.3390/jcm15145632

**Published:** 2026-07-17

**Authors:** Yiming Ding, Xiangchun Lin

**Affiliations:** Department of Gastroenterology, Peking University International Hospital, No. 1 Life Park Road, Zhongguancun Life Science Park, Changping District, Beijing 102206, China; anydym@126.com

**Keywords:** colorectal adenoma, young adults, risk inflection point, screening, Joinpoint regression, colorectal polyp risk

## Abstract

**Background/Objectives**: Early-onset colorectal cancer (EO-CRC) incidence continues to rise globally, yet age-stratified risk data for adults under 45 remain limited. **Methods**: This retrospective, single-center, colonoscopy-based cohort study included 3959 examinees aged 18–44 at Peking University International Hospital (2023–2024) and used Joinpoint regression to identify age inflection points for polyp detection rates. Case–control analysis with Least Absolute Shrinkage and Selection Operator (LASSO)-penalized logistic regression assessed metabolic risk factors for adenoma and serrated polyps in the 40–44 stratum. **Results**: Detection rates for polyps, adenomas, and serrated lesions all rose with age (*p* < 0.001). From the youngest (18–29) to the oldest (40–44) group, the polyp detection rate increased 2.6-fold, the adenoma detection rate (ADR) 4.3-fold, and the serrated lesion detection rate 2.0-fold. Joinpoint regression revealed an ADR inflection at age 33 (annual percentage change (APC): +0.51% → +1.27%), a high-risk adenoma (HRA) inflection at age 39 (APC: +0.14% → +0.77%), and an advanced-neoplasia inflection also at age 39 (APC: +0.20% → +0.81%). Across age strata, adenoma and advanced-neoplasia detection rates were comparable between asymptomatic screening and symptomatic examinees from age 35 onward (40–44 ADR 20.5% vs. 21.6%), whereas symptomatic examinees had higher rates in the youngest strata. In exploratory cross-sectional analyses within the 40–44 group, total cholesterol (odds ratio, OR = 1.47) and body mass index (BMI) (OR = 1.05) showed associations with adenoma, without establishing causality, with BMI elevation conferring risk only in women (*p* for interaction = 0.018). **Conclusions**: Adenoma risk accelerates at 33 and high-risk lesions escalate at 39, providing age-stratified risk benchmarks for adults under 45 in a predominantly symptomatic Chinese cohort. These inflection points warrant prospective validation in asymptomatic screening populations.

## 1. Introduction

Colorectal cancer (CRC) ranks as the third most common malignancy and the second leading cause of cancer-related mortality worldwide [1,2]. The burden in China is similarly substantial, with approximately 561,800 new cases and 255,700 deaths reported in 2024 [3]. Colonoscopic screening coupled with polypectomy is widely recognized as the most effective strategy for reducing CRC incidence and mortality [4,5].

For decades, major international guidelines have recommended that average-risk individuals begin CRC screening at age 50 [6]. Over the past twenty years, this approach has yielded measurable success in the over-50 population, among whom CRC incidence has stabilized or declined [7,8]. Since the early 2000s, however, early-onset CRC (EO-CRC)—defined as CRC diagnosed before age 50—has been rising globally, emerging as a pressing epidemiological concern [9,10]. The drivers of this trend are multifactorial, with evidence pointing to rising obesity, metabolic syndrome, sedentary lifestyles, and gut microbiome dysregulation [11]. Notably, EO-CRC patients experience a longer interval from symptom onset to diagnosis (median 4–6 months) than older patients [12]. Low symptom awareness among younger adults, limited clinical suspicion of malignancy in this age group, and the absence of systematic screening all contribute to delayed diagnosis, often at locally advanced or metastatic stages and with poorer outcomes [13].

Screening initiation ages differ across regions. The American Gastroenterological Association (AGA) recommends routine screening from age 45 [14], based primarily on microsimulation modeling that weighs expected benefits against screening capacity in the US population. Chinese guidelines set the threshold at 40 [15], supported by empirical data from a large-scale 2020–2024 screening program in Zhejiang Province [16] covering over 17 million examinees; Markov modeling indicated that annual screening of the 40–44 age group would maximize quality-adjusted life years and prevent the greatest number of CRC-related deaths.

Most epidemiological data have focused on the 45–49 age bracket [17], leaving the 40–44 segment—arguably the pivotal transitional window—understudied. Although EO-CRC accounts for approximately 10% of all new CRC cases, modeling projections suggest it will represent 11% of incident colon cancers and 23% of incident rectal cancers globally by 2030 [9]. Without age-stratified adenoma and polyp detection data for the 40–44 group, the specific age at which polyp risk begins to accelerate remains undefined, and the true burden of precancerous lesions cannot be accurately assessed.

Against this background, the present study conducted a large-sample retrospective analysis of a colonoscopy-based cohort using Joinpoint regression to precisely identify age inflection points for colorectal polyp risk in a Chinese population and to quantify polyp detection rates in the 40–44 age stratum, with the aim of providing objective evidence for optimizing CRC screening initiation strategies in China.

## 2. Materials and Methods

### 2.1. Study Design

This was a single-center, retrospective, observational study. Participants were stratified into four age groups: 18–29, 30–34, 35–39, and 40–44 years. The grouping rationale was as follows: (1) ages 18–29 were combined into a single interval because adenoma and polyp detection rates in this range are low, limiting the number of eligible patients with colorectal polyps; (2) from age 30 onward, detection rates begin to rise, so five-year intervals (30–34, 35–39, 40–44) were adopted to allow finer assessment of age-related risk changes. In the Joinpoint regression analysis (Joinpoint Regression Program, version 4.9.1.0; National Cancer Institute, Bethesda, MD, USA; https://surveillance.cancer.gov/joinpoint/, accessed on 14 July 2026), detection rates were calculated and analyzed by single year of age rather than by grouped intervals, ensuring more precise identification of risk acceleration inflection points.

The study was approved by the Ethics Committee of Peking University International Hospital (approval no. 2025-KY-0118-01). Owing to its retrospective design and the anonymization of all data, informed consent was waived in accordance with applicable ethical regulations.

### 2.2. Study Population

Inclusion criteria were as follows: individuals aged 18–44 who underwent colonoscopy at Peking University International Hospital between 1 January and 31 December 2024. Exclusion criteria were as follows: confirmed inflammatory bowel disease; prior history of colorectal neoplasia (polyps or malignancy); prior colorectal surgery; or incomplete examination due to inadequate bowel preparation.

Sample size was estimated using PASS 15.0 (NCSS, LLC, Kaysville, UT, USA, https://www.ncss.com/software/pass/, accessed on 14 July 2026), based on preset detection rates (8%, 14%, 18%, and 22% for the four age groups, respectively) and a non-proportional design focused on the 40–44 group (α = 0.05, power = 0.75). The required minimum sample sizes exceeded 100 for each of the 18–29, 30–34, and 35–39 groups and 500 for the 40–44 group. Because the 2024 cohort yielded insufficient eligible participants in the 18–29 range, that group was supplemented with cases from 1 January 2023 through 31 December 2024.

### 2.3. Data Collection and Definitions

Data were collected across four domains: (1) demographic information—age, sex, body mass index (BMI), lifestyle-related factors, and family history; (2) clinical information—colonoscopy indication and comorbidities; (3) pre-procedure laboratory results—metabolic and hematological parameters; (4) polyp characteristics—size range, location, and pathological findings.

The primary outcomes were colorectal polyp detection rates, expressed as the proportion of examinees with at least one lesion of a specified type. These included: overall polyp detection rate (PDR, all polyps); adenoma detection rate (ADR); serrated lesion detection rate (SDR, encompassing hyperplastic polyps, sessile serrated lesions, and traditional serrated adenomas); high-risk adenoma (HRA) detection rate—HRA defined as adenoma ≥ 10 mm in diameter, with high-grade dysplasia, or with villous component ≥ 25% (including tubulovillous and villous adenomas), excluding CRC; high-risk serrated polyp (HRS) detection rate—HRS defined as serrated polyp ≥ 10 mm or with dysplasia; and advanced-neoplasia (AN) detection rate—AN defined as the combination of HRA and HRS, excluding CRC [18,19]. This definition of advanced neoplasia (excluding CRC) was chosen to characterize precursor lesions distinctly from cancer; because some prior studies include CRC within advanced neoplasia, comparisons with such studies should account for this definitional difference.

### 2.4. Colonoscopy Quality Indicators Measurement

To ensure the credibility of detection rate findings, key colonoscopy quality indicators were reported for each age group, including the Boston Bowel Preparation Scale (BBPS) scores, cecal intubation rate, sedation method, and insertion time.

### 2.5. Statistical Analysis

Continuous variables are expressed as mean ± standard deviation and categorical variables as frequencies and percentages. Detection rates for each polyp type were calculated by age and sex stratum. Joinpoint regression was used to identify age-related inflection points, with the annual percentage change (APC) and its 95% confidence interval (CI) reported.

For the 40–44 age stratum, two independent case–control analyses were conducted, with adenoma and serrated polyp as the respective outcomes. Polyp-free controls from the 40–44 stratum were frequency-matched to the case group on sex, age (by integer year), and calendar month of colonoscopy; within each matched stratum, controls were selected at random using a computer-generated random number. A target of 300 controls was set, and 24 were subsequently excluded for excessive missing laboratory and demographic data, leaving 276 controls for analysis. The same control group was used for both analyses (i.e., controls were reused); the adenoma and serrated polyp groups partially overlapped (patients with both lesion types were included in both analyses). Consistent with the frequency-matched design, the case–control comparison used multivariable unconditional logistic regression with age and sex as covariates. Twelve metabolic and hematological candidate variables were evaluated: sex, age, BMI, fasting plasma glucose, total cholesterol, triglycerides, low-density lipoprotein cholesterol (LDL-C), uric acid, hemoglobin, alanine aminotransferase (ALT), white blood cell count, and platelet count. Each variable first underwent univariate logistic regression; bootstrap resampling (1000 repetitions) was used to obtain more robust standard errors and 95% confidence intervals. Standardized variables then entered LASSO (L1-regularized) logistic regression with five-fold cross-validation to determine the optimal regularization parameter λ (the value minimizing cross-validation error). Only variables with non-zero coefficients at this parameter were retained for multivariable logistic regression, yielding adjusted odds ratios (ORs) and 95% CIs. Because the variables entering the final model were selected by LASSO on the same data used for inference, the resulting ORs, confidence intervals, and *p* values are not corrected for this selection step and should be regarded as exploratory. The Hosmer–Lemeshow goodness-of-fit test assessed model calibration.

To evaluate the influence of examination indication, all colonoscopy examinees were classified as asymptomatic (routine health check-up) or symptomatic (lower-gastrointestinal symptoms—change in stool form or frequency, constipation, hematochezia, abdominal pain or bloating—or elevated tumor markers) using a hybrid rule: for the prospectively curated polyp cohort, the indication was taken from the recorded pre-procedure chief complaint, and for the remaining examinees the free-text indication was parsed with a standardized symptom–keyword algorithm; patients under surveillance for previously documented colorectal neoplasia and those without a documentable indication were excluded. Polyp, adenoma, high-risk adenoma, serrated lesion, high-risk serrated polyp, advanced neoplasia, and colorectal cancer detection rates were then compared between the asymptomatic and symptomatic groups across all four age strata. Sex–BMI interaction on adenoma was also examined, with sex-stratified BMI effects and the interaction *p* value reported. To assess potential temporal bias arising from the differential recruitment periods of the 18–29 subgroup, a sensitivity analysis comparing baseline characteristics and polyp-type composition between the 2023 (*n* = 41) and 2024 (*n* = 71) portions of this subgroup was performed. Additionally, to evaluate whether the age-related increase in advanced neoplasia was independent of metabolic and clinical covariates, a multivariable logistic regression for advanced neoplasia was performed among all polyp patients aged 18–44, with age, sex, BMI, total cholesterol, triglycerides, fasting glucose, hypertension, and diabetes as predictors. In addition, because colonoscopy indication was available for the full cohort, a full-cohort multivariable logistic regression with adenoma and advanced neoplasia as outcomes was performed among all first-detection examinees with a documented indication, adjusting for age, sex, and colonoscopy indication. All analyses were performed in Python 3.12 (Python Software Foundation, https://www.python.org/), with statistical significance set at *p* < 0.05. To underpin the Joinpoint analysis with transparent per-year data, for each single year of age (18–44 years), the number of examinees and detected lesions of each type, together with exact binomial 95% confidence intervals (Clopper–Pearson) for the adenoma detection rate and the advanced-neoplasia rate.

## 3. Results

### 3.1. Baseline Characteristics of the Study Population

A total of 3959 eligible colonoscopy examinees (aged 18–44) were included, of whom 1021 had colorectal polyps: 112 in the 18–29 group, 150 in the 30–34 group, 150 in the 35–39 group, and 609 in the 40–44 group. An additional 276 polyp-free controls were matched from the 40–44 stratum for risk factor analysis. Baseline characteristics of the full cohort are presented in Table 1.

Across age groups, age, BMI, and the proportion of screening indications all increased significantly (*p* < 0.05), while sex distribution remained balanced (male proportion 60–63%, *p* = 0.893). Known risk factors (smoking history, alcohol consumption, hypertension, and diabetes) and key laboratory indicators (LDL-C and fasting plasma glucose) likewise rose with age (*p* < 0.001). Polyp location (left-sided vs. right-sided colon) did not differ significantly among age groups.

### 3.2. Detection Rates of Colorectal Lesions by Age Group

Detection rates for polyps, adenomas, and serrated lesions all rose significantly with age (*p* for trend < 0.001). From the youngest group (18–29) to the oldest (40–44), the PDR increased 2.6-fold, ADR 4.3-fold, and SDR 2.0-fold (Table 2).

Sex differences in the ADR emerged from the 35–39 group onward: men had significantly higher ADR values than women in the 35–39 group (17.92% vs. 8.00%, *p* < 0.05) and the 40–44 group (22.4% vs. 16.5%, *p* < 0.001). Serrated lesion detection rates followed a similar sex-differential pattern.

HRA detection increased with age: 0.57% in the 18–29 group, 1.01% in the 30–34 group, 2.23% in the 35–39 group, and 4.59% in the 40–44 group (these group-level figures are cross-sectional prevalences; the per-year-of-age rate of change is reported separately by Joinpoint regression in Table 3). Low-risk adenoma (LRA) detection rose from 4.00% in the youngest group to 15.2% in the oldest. HRS detection remained low across all groups (0.69–1.44%), without a discernible age-related trend.

In the 40–44 group, the ADR was 19.8% and AN detection was 5.75%. Four CRC cases were identified, all in the symptomatic subgroup.

To assess potential temporal bias from pooling the 2023 and 2024 recruitment periods for the 18–29 group, detection rates were compared between the two yearly sub-cohorts (Supplementary Table S1). No significant differences were observed (all *p* > 0.05): polyp 11.5% vs. 13.7% (*p* = 0.410), adenoma 4.5% vs. 4.6% (*p* = 1.000), high-risk adenoma 0.56% vs. 0.58% (*p* = 1.000), serrated polyp 7.0% vs. 7.7% (*p* = 0.793), high-risk serrated polyp 0.56% vs. 0.77% (*p* = 1.000), and advanced neoplasia 1.13% vs. 1.35% (*p* = 1.000). These results indicated that pooling the two years is unlikely to have materially biased the 18–29 detection rates.

### 3.3. Case–Control Analysis in the 40–44 Age Stratum

Within the 40–44 group, baseline characteristics of the adenoma (*n =* 359) and serrated polyp (*n* = 270) groups were compared with frequency-matched polyp-free controls (*n* = 276) (Table 4). By design of the frequency matching, age and sex were balanced (*p* > 0.05). Adenoma patients had higher BMI than controls (25.7 vs. 25.0 kg/m^2^, *p* = 0.006); both case groups had higher proportions of inpatient origin, smoking history, and alcohol consumption (all *p* < 0.05). A smaller proportion of adenoma patients underwent colonoscopy for “changes in bowel habits” compared with controls (40.4% vs. 50.0%, *p* = 0.020), and fewer received monitored anesthesia care (91.1% vs. 96.4%, *p* = 0.012). In the serrated polyp group, fewer patients presented with “abdominal pain” than controls (1.9% vs. 5.4%, *p* = 0.045). Polyp location (left vs. right colon), prevalence of hypertension, diabetes, and family history did not differ among the three groups.

### 3.4. Joinpoint Regression Analysis

**Figure 1:**
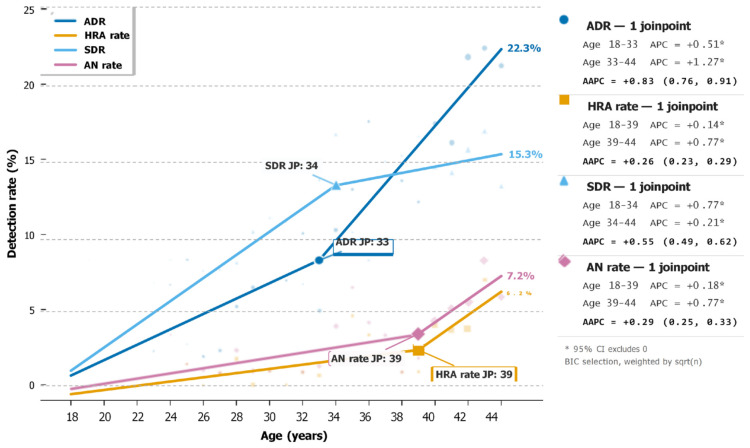
Age-specific trends in colorectal lesion detection rates among adults aged 18–44: Joinpoint regression analysis. Adenoma detection rate (ADR), with an inflection point detected at age 33; the annual percentage change (APC) accelerated from +0.51% (18–33 years) to +1.27% (33–44 years). High-risk adenoma (HRA) detection rate, with an inflection point at age 39; APC rose from +0.14% to +0.77%. Serrated lesion detection rate (SDR), with an inflection point at age 34; APC decelerated from +0.77% to +0.21%. Advanced-neoplasia (AN) detection rate, with an inflection point at age 39; APC rose from +0.20% to +0.81%. AAPC, average annual percentage change across all ages. Asterisks (*) indicate that the 95% confidence interval does not include zero. Inflection point selection was based on the Bayesian information criterion weighted by the square root of the sample size.

Joinpoint regression was applied to characterize age-specific trends in colorectal polyp detection rates among 18–44-year-olds (Figure 1, Table 3).

Overall, the ADR and SDR exhibited linear increases across the age range. The analysis revealed divergent change patterns for different lesion types.

ADR: One inflection point was detected at age 33. The APC accelerated from +0.51% (95% CI: 0.394–0.631) before 33 to +1.27% (95% CI: 1.192–1.357) after 33, with an AAPC of 0.84% (95% CI: 0.758–0.912).

HRA detection: An inflection point emerged at age 39 (ΔBIC = 9.6), where the APC surged from +0.14% (95% CI: 0.113–0.168) before 39 to +0.77% (95% CI: 0.692–0.857) after 39—a 5.5-fold increase; the AAPC = 0.26% (95% CI: 0.235–0.289).

AN detection: An inflection point likewise appeared at age 39 (ΔBIC = 2.9), with the APC rising from +0.20% (95% CI: 0.155–0.237) before 39 to +0.81% (95% CI: 0.688–0.936) after 39, a 4.1-fold increase, corroborating age 39 as a critical acceleration node for AN risk; the AAPC = 0.32% (95% CI: 0.274–0.356).

SDR: One inflection point was identified at age 34, where the APC decelerated from +0.77% to +0.21% after 34.

HRS detection: This remained linear at a very low rate across all ages (APC = +0.052%, 95% CI: 0.038–0.067), with no inflection point detected.

It should be emphasized that the age-33 and age-39 inflection points are data-derived statistical features of this single cohort rather than established biological thresholds, and their reproducibility in other populations requires external validation. The per-year-of-age sample sizes and event counts that underlie the Joinpoint analysis—including the small cell counts at the youngest ages—are reported transparently in Supplementary Table S2, which provides exact binomial 95% confidence intervals for the adenoma detection rate and the advanced-neoplasia rate at each single year of age (18–44 years).

### 3.5. Risk Factor Analysis

Univariate analysis revealed elevated total cholesterol, LDL-C, uric acid, and hemoglobin in the adenoma group (all *p* < 0.05); the serrated polyp group showed higher LDL-C, uric acid, and platelet counts.

**Figure 2:**
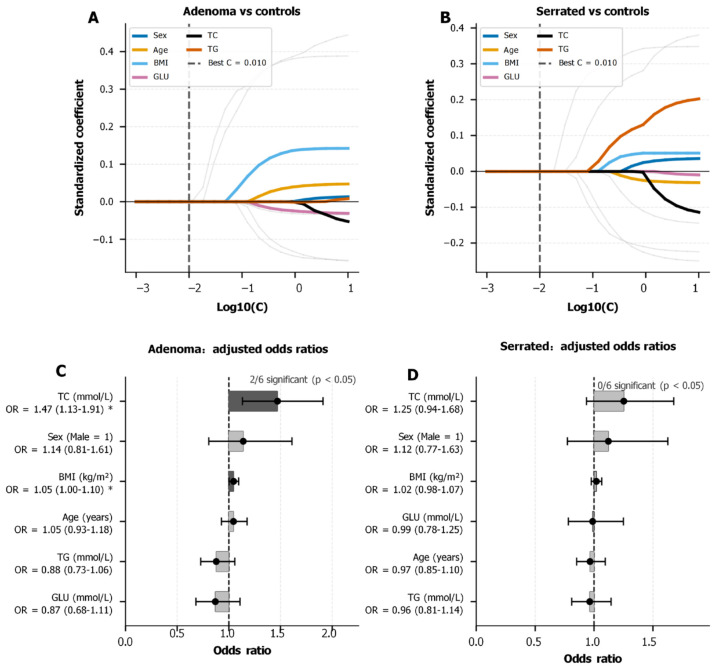
Metabolic risk factor screening and multivariable-adjusted odds ratios. (**A**,**B**) LASSO regression coefficient path plots for adenoma vs. controls (**A**) and serrated polyp vs. controls (**B**) among participants aged 40–44. Twelve clinical indicators were entered; five-fold cross-validation determined the optimal penalty parameter (λ = 0.035), and both models selected six variables: sex, age, BMI, fasting plasma glucose, total cholesterol, and triglycerides. The gray dashed vertical line in (**A**,**B**) indicates the optimal LASSO penalty parameter (λ) selected by five-fold cross-validation. (**C**,**D**) Forest plots of adjusted ORs from multivariable logistic regression for adenoma (**C**) and serrated polyp (**D**). In the adenoma model, total cholesterol (OR = 1.47, 95% CI: 1.13–1.91, *p* = 0.004) and BMI (OR = 1.05, 95% CI: 1.00–1.10, *p* = 0.045) showed the strongest exploratory associations with adenoma. No variable reached statistical significance in the serrated polyp model. * indicates statistical significance (*p* < 0.05).

LASSO regression screening of 12 clinical indicators (five-fold cross-validation, optimal λ = 0.035) selected the same six variables for both outcomes: sex, age, BMI, fasting plasma glucose, total cholesterol, and triglycerides (Figure 2A,B). In the coefficient path plots, BMI and glucose exhibited the most stable influence on adenoma, whereas triglycerides and BMI were more prominent in the serrated polyp model; age, though retained, showed rapid coefficient shrinkage due to collinearity with BMI and glucose.

Multivariable logistic regression of these six variables identified total cholesterol (OR = 1.47, 95% CI: 1.13–1.91, *p* = 0.004) and BMI (OR = 1.05, 95% CI: 1.00–1.10, *p* = 0.045) as factors independently associated with adenoma (Figure 2C). No statistically significant predictors emerged in the serrated polyp model (Figure 2D).

A significant sex–BMI interaction was observed for adenoma (*p* for interaction = 0.018) but not for HRA (*p* for interaction = 0.054) (Table 5). Sex-stratified analysis indicated that each 1 kg/m^2^ increase in BMI conferred an 8.9% higher adenoma risk in women (OR = 1.089, 95% CI: 1.024–1.158, *p* = 0.006), whereas no such association was found in men (OR = 0.994, 95% CI: 0.950–1.041, *p* = 0.799). The adenoma prediction model yielded an area under the curve (AUC) of 0.615 and the serrated polyp model an AUC of 0.568.

A multivariable analysis of advanced neoplasia and age was conducted. To assess whether age was independently associated with advanced neoplasia after adjustment for metabolic and clinical covariates, a multivariable logistic regression was performed among the 1021 polyp patients (861 with complete data; 138 advanced-neoplasia events). Age remained independently associated with advanced neoplasia after adjustment for sex, BMI, total cholesterol, triglycerides, fasting glucose, hypertension, and diabetes (OR 1.081 per year, 95% CI 1.036–1.128, *p* < 0.001; approximately 1.48 per 5 years), with the effect unchanged or slightly strengthened relative to the unadjusted estimate (OR 1.059, 95% CI 1.019–1.102). BMI (OR 1.098 per kg/m^2^, *p* = 0.001) and total cholesterol (OR 1.520 per mmol/L, *p* = 0.006) were also independently associated with advanced neoplasia (Supplementary Table S3). These findings indicate that the age-related increase in advanced lesion risk is not merely an artifact of the parallel rise in metabolic risk profile with age. In a complementary full-cohort analysis adjusting for colonoscopy indication, age likewise remained independently associated with both adenoma (OR 1.092 per year, 95% CI 1.071–1.113, *p* < 0.001) and advanced neoplasia (OR 1.091 per year, 95% CI 1.053–1.131, *p* < 0.001) after adjustment for sex and indication, with estimates essentially unchanged from the unadjusted values (adenoma 1.087; advanced neoplasia 1.088); symptomatic indication was not significantly associated with advanced neoplasia (OR 1.097, *p* = 0.631), whereas male sex was independently associated with both outcomes (adenoma OR 1.862; advanced neoplasia OR 1.766) (Supplementary Table S4). Together with the metabolic adjustment above, these analyses indicate that the age-related acceleration in lesion detection is robust to both indication mix and metabolic confounding.

### 3.6. Lesion Detection Rates by Colonoscopy Indication (Asymptomatic Screening vs. Symptomatic)

Across all four age strata, colorectal lesion detection rates were compared between asymptomatic examinees (colonoscopy for routine health check-up) and symptomatic examinees (colonoscopy for lower-GI symptoms or elevated tumor markers), using all examinees within each indication group as the denominator (Table 6). In the 35–39 and 40–44 strata, detection rates did not differ significantly between the two groups: among 40–44-year-olds, the ADR (20.49% vs. 21.55%, *p* = 0.648), HRA detection (4.93% vs. 4.54%, *p* = 0.707), advanced neoplasia (5.69% vs. 5.86%, *p* = 1.00), and CRC (0% vs. 0.38%, *p* = 0.308) were comparable, and even the asymptomatic group carried a substantial burden (ADR 20.5%, advanced neoplasia 5.7%). By contrast, in the youngest strata symptomatic examinees had significantly higher polyp and adenoma detection (18–29: ADR 2.59% vs. 9.46%, *p* = 0.017; 30–34: ADR 4.64% vs. 11.50%, *p* = 0.007). All four colorectal cancer cases occurred in symptomatic examinees aged 40–44.

### 3.7. Colonoscopy Quality Indicators

Colonoscopy quality indicators across age groups are presented in Table 7. The proportion with BBPS ≥ 6 ranged from 87.0% to 91.1%, cecal intubation rates ranged from 99.3% to 100%, and median insertion time was 3 min in all groups. These consistent quality indicators support the comparability of detection rates across age groups.

## 4. Discussion

This study systematically characterized the age distribution and temporal trends of adenoma and serrated polyp detection in a Chinese colonoscopy cohort aged 18–44. The ADR among 40–44-year-olds reached 19.8%, with HRA detection at 4.6% and the male ADR at 22.4% in this age range. Joinpoint regression identified two risk acceleration inflection points: growth in overall adenoma detection accelerated after age 33, and HRA detection surged after age 39. Sex disparities became apparent beyond age 35, with men in the 35–39 and 40–44 groups facing significantly higher adenoma risk than women of the same ages. These sex differences align with findings from most international studies [20,21,22] and may be partly attributable to smoking, alcohol consumption, and dietary preferences, underscoring the need for guidelines to account for sex-specific risk profiles.

In interpreting the age-33 inflection point, a definitive biological explanation for why adenoma growth accelerates at 33 remains elusive. One plausible hypothesis involves cumulative exposure: individuals exposed to risk factors such as smoking, alcohol use, and antibiotic consumption during early adulthood may, after years of accumulation, begin to manifest the tumor-promoting effects of these exposures on adenoma formation [23,24]. Previous research indicates that the latency from carcinogenic exposure to colorectal neoplasia typically spans several years to over a decade [25]. Yet this hypothesis cannot precisely account for why the critical inflection emerges at 33 rather than another age. The age-33 node may reflect the statistical characteristics of detection frequency trends in the present sample, and its generalizability to other populations requires validation, as the study design lacked long-term longitudinal data on risk factor exposure (e.g., initiation timing and cumulative dose). Interpretation of this inflection point therefore remains speculative, and the underlying mechanism awaits future elucidation.

Clinical significance of the age-39 inflection point. After age 39, HRA detection accelerated sharply, with the APC rising from +0.14% to +0.77%—a 5.5-fold increase. Baseline data revealed substantial clustering of metabolic risk factors in the 35–39 age range: hypertension prevalence of 10.0%, diabetes prevalence of 2.7%, and mean BMI exceeding 25 kg/m^2^. Cumulative metabolic abnormalities may accelerate the progression of adenomas to higher-grade lesions through insulin resistance, insulin-like growth factor signaling activation, and chronic low-grade inflammation [26]. It must be emphasized, however, that the cross-sectional design precludes causal inference between metabolic factors and accelerated high-risk adenoma growth; the observed association warrants further investigation.

Regarding the screening initiation age, both Chinese and US CRC screening guidelines are grounded in cost-effectiveness analyses, and their divergent age thresholds reflect legitimate differences in population context. The AGA lowered the screening start age to 45 [14], based primarily on microsimulation-predicted cost-utility ratios and existing literature; the ADR in the US 40–49 age group has been reported at approximately 11–15%. By contrast, the ADR of 19.8% we observed in 40–44-year-olds falls well above the 11–15% range reported from asymptomatic US screening cohorts. Importantly, this elevated burden was not confined to symptomatic examinees: among 40–44-year-olds, the asymptomatic screening and symptomatic groups had comparable ADRs (20.5% vs. 21.6%) and advanced-neoplasia rates (5.7% vs. 5.9%) (Table 6), indicating that the high lesion yield is not an artifact of symptomatic self-selection alone. Nevertheless, because our cohort is colonoscopy-based and predominantly symptomatic, these figures should not be interpreted as population screening yields, and the finding reinforces that adenoma risk in younger adults is no longer negligible and underscores the growing need to better characterize this age group in both countries with standardized screening cohorts. Moreover, as detailed below, part of the apparent rise in young-adult adenoma detection may reflect increased diagnostic activity rather than a pure increase in underlying risk [27], an effect our cohort cannot disentangle.

In our cohort, 40–44-year-olds carried a clinically significant polyp burden, with adenoma risk accelerating at 33 and high-risk neoplasia surging after 39. Although the cohort is predominantly symptomatic, the comparable lesion burden between asymptomatic screening and symptomatic examinees from the mid-30s onward (Table 6) indicates these observations are not driven solely by symptomatic presentation; they nonetheless align temporally with the age window targeted by the screening threshold adopted in China.

Regarding comparison with prior studies, evidence on adenoma progression risk in younger adults has been inconsistent. Early data from Martínez et al. [28] (2009), based on only 9167 patients examined before 2009, suggested lower progression risk in younger groups: using the 50–59 group as reference, the risk ratio was 0.32 for those under 40 and 0.61 for the 40–49 group—though the sample was small and the data now over 15 years old. Two larger recent studies reached different conclusions: Anderson et al. [29] (2020), using the New Hampshire Colonoscopy Registry, and Chen et al. [30] (2025), in a large prospective cohort, found that high-grade neoplasia risk in the 40–49 group was comparable to that in the 50–59 group. The shift from lower to equivalent risk parallels the global EO-CRC trend over the past two decades, suggesting that adenoma burden and progression risk in younger adults have genuinely increased over time rather than reflecting chance variation across studies.

Regarding diagnostic intensity and detection-activity effects, an important caveat is that rising adenoma and lesion detection in young adults can reflect, at least in part, increased diagnostic intensity rather than a purely biological rise in underlying risk. Recent Belgian population-based data showed that, among 40–49-year-olds, adenoma and in situ colorectal cancer rates rose over two decades in parallel with greater use of fecal immunochemical testing and colonoscopy, whereas invasive colorectal cancer incidence remained stable and mortality was stable or declined—suggesting that enhanced diagnostic activity led to earlier lesion detection rather than more advanced disease and providing no population-based evidence to support lowering the organized screening start age [27]. Our colonoscopy-based, predominantly symptomatic cohort likewise cannot disentangle a true increase in underlying lesion incidence from changes in diagnostic activity, and the Joinpoint inflection points we report describe age-specific detection within a single cohort rather than population incidence. These observations temper any interpretation of rising detection rates as direct evidence for earlier screening and underscore the need for population-based, indication-complete surveillance data.

Regarding metabolic factors and adenoma, metabolic dysfunction syndrome and its core components—overweight, hyperglycemia, and dyslipidemia—are established predisposing factors for colorectal mucosal elevated lesions [11]. The present analysis confirms associations between metabolic indicators and adenoma even in a younger population, consistent with the rising EO-CRC trend and suggesting that metabolic dysregulation may be a primary driver of the precancerous stage of EO-CRC.

The discriminative capacity of the metabolic prediction model, however, was moderate. The adenoma model achieved an adjusted AUC of 0.615, with total cholesterol and BMI as the variables most strongly associated with adenoma in this exploratory, post-selection analysis, but its individual-level predictive ability is limited and should not be interpreted as a clinically applicable risk-stratification tool. The serrated polyp model performed even less well (AUC = 0.568), with no statistically significant independent metabolic risk factors detected. These modest AUC values suggest that metabolic status may influence the development of different polyp subtypes through diverse molecular mechanisms. For instance, the BRAF mutation-driven serrated pathway tends to produce the CpG island methylator phenotype and accelerate cellular senescence, followed by epigenetic silencing of WNT pathway inhibitors—a mechanism distinct from the direct Wnt/β-catenin activation commonly seen in conventional adenomas [31,32]. Epidemiologically, the present findings support the notion that the pathogenesis of serrated polyps differs substantially from the conventional adenoma–carcinoma sequence. Future risk-stratification models for younger populations will need to incorporate additional predictive dimensions—such as fecal immunochemical test results and lifestyle factors—to improve discriminative performance [33]. Beyond baseline metabolic predictors, polyp-characteristic–based prognostic models have also been developed—for example, Facciorusso et al. [34] used recursive partitioning on adenoma size, number, and dysplasia grade to stratify post-polypectomy recurrence risk—highlighting that future risk-stratification tools for younger populations may need to integrate metabolic, lifestyle, and polyp-characteristic dimensions.

Regarding the sex–BMI interaction, in exploratory analyses, a sex–BMI interaction was observed for adenoma (*p* for interaction = 0.018); to our knowledge, this has not been previously examined in a colonoscopy-based population aged 40–44 years. Among women, higher BMI was associated with increased adenoma risk (OR per 1 kg/m^2^ = 1.089, 95% CI: 1.024–1.158, *p* = 0.006), whereas no association was evident in men. Although nominally significant, these subgroup findings should be interpreted with considerable caution. The main effect of BMI in the overall cohort was marginal (OR = 1.05, 95% CI: 1.00–1.10, *p* = 0.045), and the modest effect size limits clinical interpretation at the individual level. The observed interaction, while statistically significant, is exploratory in nature and requires confirmation in independent cohorts. These results raise the hypothesis that obesity could carry a stronger adenoma risk signal in younger women than in younger men, but this notion remains speculative and warrants prospective evaluation.

Regrading public health implications of the two inflection points, acceleration of adenoma risk at age 33 implies that primary prevention—including weight management, dietary modification, and smoking cessation—should begin well before 40 and that clinicians should maintain a low threshold for colonoscopy in patients in their thirties presenting with gastrointestinal symptoms. The sharp increase in high-risk adenoma after age 39 is broadly compatible with the heightened vigilance around age 40 reflected in current Chinese screening recommendations; however, because these observations derive from a predominantly symptomatic, single-center cohort, they constitute hypothesis-generating rather than policy-grade evidence and cannot by themselves determine the optimal population screening age. Integrating these findings, a two-stage strategy may be worth future evaluation—primary prevention and clinical alertness beginning in the early thirties, with systematic screening commencing around the age window indicated by the HRA inflection (i.e., approximately 40 years)—an approach broadly aligned with the prevailing screening threshold in China, although requiring confirmation in asymptomatic screening cohorts. Future guidelines should evaluate whether men require earlier or more intensive screening. These implications are hypothesis-generating; the symptomatic, single-center, retrospective design precludes direct inference about optimal population screening initiation, which requires prospective validation in asymptomatic, multicenter cohorts.

Independence of the age effect from metabolic confounding also must be noted. Because BMI, metabolic, and lifestyle risk factors rise with age (Table 1), the observed age trend in lesion detection could partly reflect confounding by the metabolic profile rather than age itself. To address this, a multivariable analysis among polyp patients (Supplementary Table S3) showed that age remained independently associated with advanced neoplasia after adjustment for BMI, cholesterol, triglycerides, glucose, hypertension, and diabetes (OR 1.081 per year), indicating that the age-related acceleration of advanced-lesion risk is not merely a proxy for the rising metabolic risk profile. We note, however, that this is a within-polyp-patient analysis of lesion severity and cannot fully replace a population-level detection analysis, which was precluded by the limited availability of metabolic data across the full cohort.

Adenoma detection versus cancer risk should also be addressed. This study characterizes colorectal precursor lesions; only four colorectal cancer cases were identified, all in symptomatic patients. The adenoma and advanced-neoplasia inflection points therefore describe the age distribution of detectable precursor lesions in this cohort and should not be interpreted as directly explaining early-onset colorectal cancer incidence or mortality trends. It is important to distinguish adenoma detection, advanced-neoplasia detection, and actual cancer risk: the present findings are relevant to prevention and early detection, but lesion-progression risk and cancer outcomes cannot be established without longitudinal follow-up.

The strengths of this research are as follows. One methodological strength of this study lies in applying Joinpoint regression to characterize colorectal polyp prevalence in a young Chinese population, enabling objective and quantitative identification of the specific age at which risk increases. All polyps were confirmed by pathological examination, ensuring diagnostic accuracy. Power analysis indicated that, given the current sample size and observed effect sizes, the statistical power for detecting differences in main lesion detection rates across age groups exceeded 99.9% (α = 0.05), providing robust statistical support for the inflection points at ages 33 and 39 and the observed sex differences. Additionally, colonoscopy quality indicators were reported and a sensitivity analysis comparing screening and symptomatic subgroups was conducted to assess the potential impact of selection bias.

Several limitations should be acknowledged. First, as a single-center retrospective study conducted at a tertiary-care hospital, the included cases were predominantly symptomatic patients who sought care proactively; this design may introduce selection bias. Only approximately 29% of examinees in the 40–44 group (527 of 1810) underwent colonoscopy for routine physical examination, and this proportion was lower in the youngest group (13.3% for 18–29 years, 116 of 874). Consequently, the reported polyp detection rates and risk inflection points may overestimate the true burden of colorectal polyps in the general young population, and the findings may have limited generalizability to other geographic regions, healthcare settings, and population structures, as well as to asymptomatic screening populations. A direct comparison of asymptomatic screening and symptomatic examinees across all age strata (Table 6), however, showed no significant differences in adenoma, high-risk adenoma, advanced neoplasia, or colorectal cancer detection in the 35–39 and 40–44 groups (e.g., 40–44 ADR 20.5% vs. 21.6%), indicating that from the mid-30s onward the indication mix did not materially alter the within-cohort lesion profile; in the youngest strata, detection was significantly higher among symptomatic examinees, consistent with the very low baseline burden of asymptomatic adults under 35. Second, although the sample size is relatively large for a study of the 18–44 age group, it remains limited compared with multicenter studies involving the broader participation. Third, the primary data were collected during a single calendar year (2024), and this limited time span introduces potential concerns: polyp detection rates can vary year to year due to factors such as endoscopist experience and equipment upgrades, and a single-year dataset cannot adequately capture historical trends. The Joinpoint-derived age inflection points at 33 and 39 years were identified within this single dataset and have not been externally validated; their reproducibility in other Chinese or international populations remains to be determined. Multi-year longitudinal studies are needed to monitor temporal trends in detection rates and the stability of inflection point ages. Fourth, there is a risk of selection bias arising from inconsistent time frames: because colorectal polyp incidence in the 18–29 age group is extremely low, data from both 2023 and 2024 were included to ensure adequate statistical power, whereas groups aged 30 and above included only 2024 data. This difference in temporal coverage may introduce temporal bias. A sensitivity analysis, however, showed no significant differences between the 2023 and 2024 portions of this subgroup in baseline characteristics or polyp-type composition (Supplementary Table S1), indicating that pooling the two years is unlikely to have materially biased the results. Fifth, the discriminative capacity of the metabolic prediction models was limited (adenoma AUC = 0.615; serrated polyp AUC = 0.568), which is insufficient for individual-level clinical risk stratification. Sixth, owing to the cross-sectional design, metabolic exposures (BMI, total cholesterol) and adenoma outcomes were assessed simultaneously, precluding causal or temporal inference; the reported associations should be interpreted as cross-sectional associations rather than causal effects. Seventh, several established colorectal neoplasia risk factors—including dietary patterns, physical activity, socioeconomic status, medication use (e.g., aspirin, statins), genetic predisposition, and gut microbiome composition—were not routinely captured and could not be adjusted for; residual confounding from these unmeasured variables cannot be excluded. Eighth, the LASSO-selected metabolic risk factor model is exploratory: because variable selection and inference were performed on the same data, the odds ratios, confidence intervals, and *p* values are not corrected for the selection step and should be interpreted as hypothesis-generating. Ninth, Table 1 and Table 3 involve a large number of unadjusted comparisons that were not corrected for multiplicity; these analyses are intended to be descriptive, and borderline findings should be interpreted with caution (notably the sex × BMI interaction, which is exploratory and rests on a modest number of obese women). Tenth, controls were required to have complete metabolic and laboratory data; although frequency-matched on sex, age, and calendar month, this requirement could introduce selection bias if data completeness is associated with adenoma status. BMI and metabolic laboratory values were available only for the case–control analysis sample (polyp patients and the selected controls) and not for the broader examinee cohort, so BMI-stratified population prevalences could not be reported. Eleventh, a full-cohort multivariable adjustment of the age–lesion association for metabolic and lifestyle confounders was not possible because BMI, metabolic, smoking, and comorbidity data were available only for the case–control analysis sample and not for the broader examinee cohort; the multivariable analysis among polyp patients (Supplementary Table S3) provides partial, within-sample evidence but cannot fully replace a population-level analysis. Twelfth, the same control group was used for both the adenoma and serrated polyp case–control analyses, and the adenoma and serrated case groups partially overlapped; consequently, these two analyses are not statistically independent, which is acknowledged as a limitation. Thirteenth, rising lesion detection rates observed in this colonoscopy-based cohort may partly reflect temporal changes in diagnostic activity (e.g., expanding colonoscopy and fecal immunochemical test use) rather than a pure increase in underlying incidence, an effect documented in recent population-based data [27]; our single-center study design cannot separate these contributions. Fourteenth, withdrawal time—the colonoscopy quality indicator most directly linked to adenoma detection—was not systematically captured as a discrete field in our institution’s reporting system, which records insertion/cecal intubation time but not withdrawal time; we therefore could not adjust detection rates for withdrawal time, and its variability represents an unmeasured source of detection heterogeneity.

## 5. Conclusions

This retrospective, single-center, colonoscopy-based cohort study provides a systematic characterization of the age distribution of colorectal polyps in a predominantly symptomatic Chinese cohort aged 18–44, suggesting that adenoma risk may begin accelerating around age 33 and high-risk adenoma risk around age 39. These findings provide hypothesis-generating evidence relevant to the CRC screening initiation age in China but require prospective validation in asymptomatic, multicenter populations before informing guideline changes. Men in this age range, particularly those with metabolic risk factors, may warrant priority clinical attention.

## Figures and Tables

**Table 1:** Baseline characteristics of patients with colorectal polyps, stratified by age group.

Variable	18–29 y (*n* = 112)	30–34 y (*n* = 150)	35–39 y (*n* = 150)	40–44 y (*n* = 609)	*p* Value
Demographics					
Age (years, mean ± SD)	27.5 ± 2.1	33.1 ± 1.5	37.7 ± 1.4	42.6 ± 1.4	—
Sex (male, %)	70 (62.5%)	91 (60.7%)	94 (62.7%)	364 (59.8%)	0.893
BMI (kg/m^2^, mean ± SD)	24.3 ± 4.8	24.8 ± 4.1	25.4 ± 4.1	25.6 ± 3.7	0.002
Clinical History					
Inpatient source (%)	10 (8.9%)	16 (10.7%)	22 (14.7%)	95 (15.6%)	0.163
Painless anesthesia (%)	95 (84.8%)	142 (94.7%)	140 (93.3%)	565 (92.8%)	0.016
Family history (%)	6 (5.4%)	8 (5.3%)	6 (4.0%)	27 (4.4%)	0.921
Endoscopy Indications					
Health check-up (%)	5 (4.5%)	31 (20.7%)	46 (30.7%)	184 (30.2%)	<0.001
Abdominal pain (%)	18 (16.1%)	8 (5.3%)	8 (5.3%)	33 (5.4%)	<0.001
Bloating (%)	12 (10.7%)	15 (10.0%)	10 (6.7%)	32 (5.3%)	0.058
Bowel habit change (%)	61 (54.5%)	72 (48.0%)	74 (49.3%)	271 (44.5%)	0.220
Hematochezia/melena (%)	16 (14.3%)	18 (12.0%)	6 (4.0%)	46 (7.6%)	0.008
Other indications (%)	0 (0.0%)	6 (4.0%)	6 (4.0%)	43 (7.1%)	0.013
Polyp Location					
Left colon (%)	64 (57.1%)	78 (52.0%)	73 (48.7%)	319 (52.4%)	0.603
Right colon (%)	52 (46.4%)	66 (44.0%)	77 (51.3%)	320 (52.5%)	0.227
Lifestyle & Comorbidities					
Smoking history (%)	1 (0.9%)	8 (5.3%)	8 (5.3%)	71 (11.7%)	<0.001
Alcohol history (%)	3 (2.7%)	9 (6.0%)	12 (8.0%)	89 (14.6%)	<0.001
Hypertension (HTN, %)	3 (2.7%)	4 (2.7%)	15 (10.0%)	101 (16.6%)	<0.001
Diabetes (DM, %)	1 (0.9%)	1 (0.7%)	4 (2.7%)	40 (6.6%)	0.001
Fatty liver (NAFLD, %)	15 (13.4%)	27 (18.0%)	33 (22.0%)	137 (22.5%)	0.130
Laboratory Tests					
ALT (U/L)	26.9 ± 28.4	26.4 ± 20.2	26.3 ± 17.1	23.6 ± 13.4	0.714
AST (U/L)	24.4 ± 15.5	23.5 ± 7.9	23.4 ± 9.1	25.0 ± 7.3	<0.001
GGT (U/L)	22.8 ± 17.0	27.6 ± 31.9	29.6 ± 23.6	22.7 ± 20.7	<0.001
LDL (mmol/L)	2.8 ± 0.7	2.8 ± 0.6	3.0 ± 0.6	2.8 ± 0.7	<0.001
Total cholesterol (mmol/L)	4.5 ± 0.7	4.5 ± 0.7	4.7 ± 0.7	4.5 ± 0.8	<0.001
Triglycerides (mmol/L)	1.2 ± 0.9	1.3 ± 1.0	1.4 ± 1.0	1.3 ± 1.2	0.005
Uric acid (μmol/L)	418.2 ± 98.8	400.7 ± 104.5	399.9 ± 113.3	420.0 ± 98.3	0.035
Fasting glucose (mmol/L)	5.4 ± 0.9	5.4 ± 0.6	5.4 ± 0.8	5.7 ± 0.9	<0.001
Eosinophils (%)	3.0 ± 2.5	3.1 ± 2.1	2.4 ± 1.8	3.6 ± 2.2	<0.001

Data are presented as mean ± SD or *n* (%). *p* values were calculated using One-way ANOVA for continuous variables and Chi-square test for categorical variables.

**Table 2:** Prevalence of colorectal lesions by age and sex among adults aged 18–44 years undergoing colonoscopy.

Age Group, y	Sex	Total Patients, No.	Prevalence, No. (%)				
			Adenoma	HRA	Serrated Polyps	HRS	AN
18–29	Both	874	40 (4.58)	5 (0.57)	65 (7.43)	6 (0.69)	11 (1.26)
	Male	428	22 (5.14)	4 (0.93)	38 (8.88)	3 (0.70)	7 (1.64)
	Female	446	18 (4.04)	1 (0.22)	27 (6.05)	3 (0.67)	4 (0.90)
30–34	Both	693	60 (8.66)	7 (1.01)	87 (12.6)	5 (0.72)	12 (1.73)
	Male	339	38 (11.2)	3 (0.88)	52 (15.3)	2 (0.59)	5 (1.47)
	Female	354	22 (6.21)	4 (1.13)	35 (9.89)	3 (0.85)	7 (1.98)
35–39	Both	582	77 (13.2)	13 (2.23)	75 (12.9)	7 (1.20)	19 (3.26)
	Male	307	55 (17.92)	10 (3.26)	44 (14.3)	5 (1.63)	15 (4.89)
	Female	275	22 (8.00)	3 (1.09)	31 (11.3)	2 (0.73)	4 (1.45)
40–44	Both	1810	359 (19.8)	83 (4.59)	270 (14.9)	26 (1.44)	104 (5.75)
	Male	1012	227 (22.4)	57 (5.63)	166 (16.40)	16 (1.58)	68 (6.72)
	Female	798	132 (16.5)	26 (3.26)	104 (13.0)	10 (1.25)	36 (4.51)

Data are presented as *n* (%). Note: high-risk adenoma (HRA) was defined as an adenoma with a diameter of 10 mm or greater, high-grade dysplasia, or villous components. High-risk serrated polyp (HRS) was defined as a serrated polyp 10 mm or greater or with dysplasia. Advanced neoplasia (AN) was defined as the presence of HRA, HRS.

**Table 3:** Joinpoint regression analysis of age-specific trends in colorectal lesion detection rates among adults aged 18–44 years.

Variable	No. of Joinpoints	Joinpoint (Age, Years)	Segment (Age Range)	APC (95% CI)	AAPC (95% CI)	ΔBIC
PDR	0	—	18–44	1.440 (1.395, 1.485) *	1.44 (1.40, 1.49)	0.00
ADR	1	33	18–33	0.512 (0.394, 0.631) *	0.835 (0.758, 0.912)	4.85
			33–44	1.275 (1.192, 1.357) *		
HRA rate	1	39	18–39	0.140 (0.113, 0.168) *	0.262 (0.235, 0.289)	9.59
			39–44	0.774 (0.692, 0.857) *		
SDR	1	34	18–34	0.770 (0.679, 0.862) *	0.554 (0.490, 0.618)	1.77
			34–44	0.207 (0.128, 0.286) *		
HRS rate	0	—	18–44	0.052 (0.038, 0.067) *	0.052 (0.038, 0.067)	0.00
AN rate	1	39	18–39	0.196 (0.155, 0.237) *	0.315 (0.274, 0.356)	2.93
			39–44	0.812 (0.688, 0.936) *		

APC, annual percent change; AAPC, average annual percent change; CI, confidence interval; BIC, Bayesian information criterion; PDR, polyp detection rate; ADR, adenoma detection rate; HRA, high-risk adenoma; SDR, serrated polyp detection rate; HRS, high-risk serrated polyp; AN, advanced neoplasia. * Indicates that the 95% CI of the APC does not include zero (i.e., the trend is statistically significant). ΔBIC values represent the difference in BIC compared to the model with 0 Joinpoints.

**Table 4:** Comparison of baseline characteristics among the control, adenoma, and serrated polyp groups in the 40–44 years age stratum.

Variable	Control (≥40 y, *n* = 276)	Adenoma (≥40 y, *n* = 359)	Serrated (≥40 y, *n* = 270)	*p* (Ctrl vs. Ade)	*p* (Ctrl vs. Ser)	*p* (Ade vs. Ser)
Demographics						
Age (years, mean ± SD)	42.6 ± 1.4	42.7 ± 1.4	42.5 ± 1.4	0.608	0.562	0.264
Sex (male, %)	162 (58.7%)	226 (63.0%)	170 (63.0%)	0.313	0.351	1.000
BMI (kg/m^2^, mean ± SD)	25.0 ± 4.6	25.7 ± 3.7	25.5 ± 3.8	0.006 ^b^	0.114	0.291
Clinical History						
Inpatient source (%)	7 (2.5%)	70 (19.5%)	40 (14.8%)	<0.001 ^b^	<0.001 ^d^	0.154
Painless anesthesia (%)	266 (96.4%)	327 (91.1%)	254 (94.1%)	0.012 ^a^	0.288	0.213
Family history (%)	14 (5.1%)	22 (6.1%)	7 (2.6%)	0.691	0.199	0.057
Endoscopy Indications						
Health check-up (%)	73 (26.4%)	116 (32.3%)	74 (27.4%)	0.130	0.876	0.216
Abdominal pain (%)	15 (5.4%)	28 (7.8%)	5 (1.9%)	0.309	0.045 ^c^	0.002 ^f^
Bloating (%)	13 (4.7%)	21 (5.8%)	9 (3.3%)	0.649	0.548	0.202
Bowel habit change (%)	138 (50.0%)	145 (40.4%)	137 (50.7%)	0.020 ^a^	0.930	0.012 ^e^
Hematochezia/melena (%)	21 (7.6%)	25 (7.0%)	27 (10.0%)	0.876	0.403	0.222
Other indications (%)	16 (5.8%)	24 (6.7%)	18 (6.7%)	0.770	0.808	1.000
Polyp Location						
Left colon (%)	—	207 (57.7%)	148 (54.8%)	—	—	0.528
Right colon (%)	—	201 (56.0%)	146 (54.1%)	—	—	0.691
Lifestyle & Comorbidities						
Smoking history (%)	14 (5.1%)	49 (13.6%)	34 (12.6%)	<0.001 ^b^	0.003 ^d^	0.788
Alcohol history (%)	24 (8.7%)	56 (15.6%)	41 (15.2%)	0.013 ^a^	0.027 ^c^	0.976
Hypertension (HTN, %)	37 (13.4%)	62 (17.3%)	46 (17.0%)	0.222	0.288	1.000
Diabetes (DM, %)	17 (6.2%)	22 (6.1%)	15 (5.6%)	1.000	0.906	0.896
Fatty liver (NAFLD, %)	63 (22.8%)	91 (25.3%)	50 (18.5%)	0.521	0.256	0.053
Laboratory Tests						
ALT (U/L)	24.2 ± 11.7	24.3 ± 15.3	22.5 ± 9.4	0.115	0.028 ^c^	0.463
AST (U/L)	25.9 ± 6.2	25.1 ± 7.8	24.7 ± 6.1	0.018 ^a^	0.047 ^c^	0.765
GGT (U/L)	23.5 ± 24.1	23.9 ± 21.9	22.1 ± 21.1	0.313	0.892	0.326
LDL (mmol/L)	2.7 ± 0.6	2.9 ± 0.7	2.8 ± 0.6	<0.001 ^b^	0.015 ^c^	0.106
Total cholesterol (mmol/L)	4.4 ± 0.8	4.6 ± 0.8	4.5 ± 0.7	<0.001 ^b^	0.045 ^c^	0.200
Triglycerides (mmol/L)	1.2 ± 1.2	1.2 ± 0.9	1.3 ± 1.4	0.002 ^b^	0.033 ^c^	0.355
Uric acid (μmol/L)	392.0 ± 75.4	428.4 ± 98.4	425.3 ± 93.2	<0.001 ^b^	<0.001 ^d^	0.918
Fasting glucose (mmol/L)	5.8 ± 0.6	5.7 ± 0.8	5.7 ± 0.9	0.001 ^b^	0.105	0.157
Eosinophils (%)	3.8 ± 2.2	3.5 ± 1.9	3.9 ± 2.5	0.326	0.747	0.149

Data are presented as mean ± SD or *n* (%). *p* values were calculated using independent *t*-tests for continuous variables and Chi-square tests for categorical variables. Statistical significance: ^a^ *p* < 0.05, ^b^ *p* < 0.01 control vs. adenoma; ^c^ *p* < 0.05, ^d^ *p* < 0.01 control vs. serrated; ^e^ *p* < 0.05, ^f^ *p* < 0.01 adenoma vs. serrated.

**Table 5:** Sex-specific association between body mass index and the presence of adenoma, with interaction analysis.

Model/Stratification	Variable	Category/Unit	Male	Female	*p* for Interaction
Sex × BMI interaction	Adenoma (yes/no)	—	—	—	0.018
	HRA (yes/no)	—	—	—	0.054
Sex-stratified BMI effect (continuous)	Adenoma	Per 1 kg/m^2^ increase	0.994 (0.950–1.041)	1.089 (1.024–1.158)	—
		*p* value	0.799	0.006	

Data are presented as odds ratio (95% confidence interval) for continuous BMI. HRA, high-risk adenoma. *p* for interaction was derived from logistic regression models including sex, BMI, and their product term.

**Table 6:** Comparison of colorectal lesion detection rates between asymptomatic (health check-up) and symptomatic examinees, by age stratum, among adults aged 18–44 years undergoing colonoscopy.

Age Stratum	Indication	*n*	PDR	ADR	HRA	SDR	HRS	AN	AN + CRC	CRC
18–29	Asymptomatic	116	5 (4.31)	3 (2.59)	1 (0.86)	2 (1.72)	0 (0.00)	1 (0.86)	1 (0.86)	0 (0.00)
	Symptomatic	391	105 (26.85)	37 (9.46)	4 (1.02)	63 (16.11)	6 (1.53)	10 (2.56)	10 (2.56)	0 (0.00)
		*p* value	<0.001 ***	0.017 *	1.000	<0.001 ***	0.345	0.470	0.470	1.000
30–34	Asymptomatic	194	30 (15.46)	9 (4.64)	2 (1.03)	21 (10.82)	2 (1.03)	4 (2.06)	4 (2.06)	0 (0.00)
	Symptomatic	426	116 (27.23)	49 (11.50)	5 (1.17)	64 (15.02)	3 (0.70)	8 (1.88)	8 (1.88)	0 (0.00)
		*p* value	0.001 **	0.007 **	1.000	0.168	0.651	1.000	1.000	1.000
35–39	Asymptomatic	171	44 (25.73)	22 (12.87)	4 (2.34)	21 (12.28)	2 (1.17)	6 (3.51)	6 (3.51)	0 (0.00)
	Symptomatic	335	103 (30.75)	52 (15.52)	9 (2.69)	54 (16.12)	6 (1.79)	13 (3.88)	13 (3.88)	0 (0.00)
		*p* value	0.256	0.506	1.000	0.291	0.723	1.000	1.000	1.000
40–44	Asymptomatic	527	175 (33.21)	108 (20.49)	26 (4.93)	71 (13.47)	5 (0.95)	30 (5.69)	30 (5.69)	0 (0.00)
	Symptomatic	1058	406 (38.37)	228 (21.55)	48 (4.54)	187 (17.67)	18 (1.70)	62 (5.86)	66 (6.24)	4 (0.38)
		*p* value	0.046 *	0.648	0.707	0.036 *	0.273	1.000	0.738	0.308

*p* values by two-sided Fisher’s exact test comparing asymptomatic vs. symptomatic examinees within each age stratum (* *p* < 0.05, ** *p* < 0.01, *** *p* < 0.001). Values are number of examinees with the lesion (detection rate, %). PDR, polyp detection rate; ADR, adenoma detection rate; HRA, high-risk adenoma; SDR, serrated lesion detection rate; HRS, high-risk serrated polyp; AN, advanced neoplasia (HRA + HRS, excluding CRC). Asymptomatic = colonoscopy for routine health check-up; symptomatic = colonoscopy for lower-gastrointestinal symptoms (change in stool form or frequency, constipation, hematochezia, abdominal pain or bloating) or elevated tumor markers. Patients undergoing surveillance for previously documented colorectal neoplasia (per the pre-specified exclusion criterion) and examinees without a documentable indication were excluded from the indication-stratified comparison. All four colorectal cancer cases occurred in symptomatic examinees aged 40–44.

**Table 7:** Colonoscopy quality indicators by age group.

Group	BBPS Available, *n*	BBPS ≥ 6, *n* (%)	BBPS, Median (IQR)	Cecal Intubation, *n* (%)	Conscious Sedation, *n* (%)	Insertion Time Available, *n*	Insertion Time, Median (IQR), min
18–29 y (*n* = 112)	108	94 (87.0)	7 (6–8)	112 (100)	95 (84.8)	110	3 (2–5)
30–34 y (*n* = 150)	149	132 (88.6)	7 (6–8)	150 (100)	142 (94.7)	149	3 (2–4)
35–39 y (*n* = 150)	147	129 (87.8)	7 (6–8)	150 (100)	140 (93.3)	148	3 (2–4)
40–44 y (*n* = 609)	595	542 (91.1)	7 (6–8)	605 (99.3)	565 (92.8)	605	3 (2–5)
Controls (*n* = 276)	271	247 (91.1)	7 (6–8)	276 (100)	266 (96.4)	275	3 (2–5)

Abbreviations: BBPS = Boston Bowel Preparation Scale; IQR = interquartile range. Adequate bowel preparation was defined as BBPS ≥ 6. Cecal intubation was defined as documentation of reaching the cecum or terminal ileum. Insertion time was recorded from anal insertion to cecal intubation. All quality indicators were extracted from the standardized colonoscopy reporting system at Peking University International Hospital.

## Data Availability

The data presented in this study are available on request from the corresponding author. The data are not publicly available due to privacy and ethical restrictions.

## Supplementary Materials

The following supporting information can be downloaded at https://www.mdpi.com/article/10.3390/jcm15145632/s1, Table S1: Comparison of polyp and lesion detection rates and baseline characteristics of polyp patients between the 2023 and 2024 recruitment sub-cohorts of 18–29-year-old examinees; Table S2: Number of colonoscopy examinees and detected lesions by single year of age (18–44 years), with exact binomial 95% confidence intervals for the adenoma detection rate (ADR) and the advanced-neoplasia rate; Table S3: Multivariable logistic regression for advanced neoplasia among polyp patients aged 18–44; Table S4: Full-cohort multivariable logistic regression for adenoma and advanced neoplasia among first-detection colonoscopy examinees (asymptomatic screening + symptomatic), adjusting for age, sex, and colonoscopy indication.

## Abbreviations

The following abbreviations are used in this manuscript:

EO-CRC	early-onset colorectal cancer
CRC	colorectal cancer
ADR	adenoma detection rate
PDR	polyp detection rate
SDR	serrated lesion detection rate
HRA	high-risk adenoma
HRS	high-risk serrated polyp
AN	advanced neoplasia
LRA	low-risk adenoma
APC	annual percentage change
AAPC	average annual percentage change
BMI	body mass index
LDL-C	low-density lipoprotein cholesterol
ALT	alanine aminotransferase
BBPS	Boston Bowel Preparation Scale
OR	odds ratio
CI	confidence interval
AUC	area under the curve
ROC	receiver operating characteristic
AGA	American Gastroenterological Association
LASSO	least absolute shrinkage and selection operator
