# Supplementary material for: Age Inflection Points of Colorectal Adenoma Risk in Young Adults: A Joinpoint Regression Analysis of a Single-Center Retrospective Colonoscopy-Based Cohort
Source: J Clin Med. 2026 Jul 17;15(14):5632. doi: 10.3390/jcm15145632 (PMC13413032; doi:10.3390/jcm15145632)
Supplement: Supplementary file 1 [file jcm-15-05632-s001.zip › jcm-4418719-supplementary.pdf]

Supplementary Tables

Supplementary Tables S1–S4 for: “Age Inflection Points of Colorectal Adenoma Risk in Young Adults: A Joinpoint Regression Analysis” (Manuscript ID: jcm-4418719).

Supplementary Table S1. Comparison of polyp and lesion detection rates, and baseline characteristics of polyp patients, between the 2023 and 2024 recruitment sub-cohorts of 18–29-year-old examinees.

Variable	2023(n = 355)	2024(n = 519)	P value
Polyp	41 (11.5)	71 (13.7)	0.410 [b]
Adenoma	16 (4.5)	24 (4.6)	1.000 [b]
High-risk adenoma	2 (0.56)	3 (0.58)	1.000 [b]
Serrated polyp	25 (7.0)	40 (7.7)	0.793 [b]
High-risk serrated polyp	2 (0.56)	4 (0.77)	1.000 [b]
Advanced neoplasia	4 (1.13)	7 (1.35)	1.00 [b]
Age, years, mean ± SD	27.7 ± 1.9	27.2 ± 2.3	0.33 [a]
Male sex, n (%)	24 (58.5)	46 (64.8)	0.55 [b]
BMI, kg/m², mean ± SD	23.3 ± 5.2	24.8 ± 4.6	0.06 [a]
Family history of polyp/CRC, n (%)	4 (9.8)	2 (2.8)	0.19 [b]
Smoking, n (%)	1 (2.4)	0 (0.0)	0.37 [b]
Drinking, n (%)	2 (4.9)	1 (1.4)	0.55 [b]

[a] Mann–Whitney U test; [b] Fisher's exact test. Detection rates are expressed per eligible examinee (denominators: all eligible 18–29 examinees, n = 355 in 2023 and 519 in 2024; pooled n = 874). Baseline characteristics refer to the polyp-patient subgroups (n = 41 and 71). BMI available for 32 (2023) and 64 (2024) polyp patients owing to missing height/weight. All comparisons non-significant (P > 0.05).

Supplementary Table S2. Number of colonoscopy examinees and detected lesions by single year of age (18–44 years), with exact binomial 95% confidence intervals for the adenoma detection rate (ADR) and the advanced-neoplasia rate.

Age	n	Polyps	Adenoma	HRA	Serrated	HRS	AN	CRC	ADR % (95% CI)	AN % (95% CI)
18	9	0	0	0	0	0	0	0	0.00% (0.0–33.6)	0.00% (0.0–33.6)
19	12	1	1	0	0	0	0	0	8.33% (0.2–38.5)	0.00% (0.0–26.5)
20	10	0	0	0	0	0	0	0	0.00% (0.0–30.8)	0.00% (0.0–30.8)
21	17	1	0	0	1	0	0	0	0.00% (0.0–19.5)	0.00% (0.0–19.5)
22	27	1	0	0	1	0	0	0	0.00% (0.0–12.8)	0.00% (0.0–12.8)
23	44	5	1	0	3	0	0	0	2.27% (0.1–12.0)	0.00% (0.0–8.0)
24	60	9	4	0	4	2	2	0	6.67% (1.8–16.2)	3.33% (0.4–11.5)
25	72	10	6	1	2	0	1	0	8.33% (3.1–17.3)	1.39% (0.0–7.5)
26	105	12	2	0	8	1	1	0	1.90% (0.2–6.7)	0.95% (0.0–5.2)
27	130	16	3	1	10	0	1	0	2.31% (0.5–6.6)	0.77% (0.0–4.2)
28	172	21	9	3	14	1	4	0	5.23% (2.4–9.7)	2.33% (0.6–5.8)
29	216	36	14	0	22	2	2	0	6.48% (3.6–10.6)	0.93% (0.1–3.3)
30	108	21	11	1	12	0	1	0	10.19% (5.2–17.5)	0.93% (0.0–5.1)
31	101	17	7	0	11	0	0	0	6.93% (2.8–13.8)	0.00% (0.0–3.6)
32	119	20	13	1	8	0	1	0	10.92% (5.9–18.0)	0.84% (0.0–4.6)
33	161	36	8	1	22	1	2	0	4.97% (2.2–9.6)	1.24% (0.2–4.4)
34	204	56	21	4	34	4	8	0	10.29% (6.5–15.3)	3.92% (1.7–7.6)
35	108	26	14	5	11	3	7	0	12.96% (7.3–20.8)	6.48% (2.6–12.9)

36	120	31	21	4	10	1	5	0	17.50% (11.2–25.5)	4.17% (1.4–9.5)
37	121	27	12	2	17	3	4	0	9.92% (5.2–16.7)	3.31% (0.9–8.2)
38	118	33	16	1	18	0	1	0	13.56% (8.0–21.1)	0.85% (0.0–4.6)
39	115	33	14	1	19	1	2	0	12.17% (6.8–19.6)	1.74% (0.2–6.1)
40	351	111	60	13	51	3	16	1	17.09% (13.3–21.4)	3.99% (2.2–6.6)
41	353	102	57	13	51	6	17	1	15.86% (12.2–20.1)	4.82% (2.8–7.6)
42	455	163	99	18	69	8	24	1	21.54% (17.8–25.6)	5.27% (3.4–7.7)
43	328	114	71	18	49	6	23	1	21.65% (17.5–26.7)	7.01% (4.8–10.8)
44	323	119	72	20	50	3	23	0	22.29% (18.3–27.6)	7.16% (3.3–8.6)

HRA, high-risk adenoma; HRS, high-risk serrated polyp; AN, advanced neoplasia (HRA + HRS); ADR, adenoma detection rate. Single-year estimates at the youngest ages (18–22 years) rest on small numbers of examinees and events and should be interpreted with caution. Confidence intervals by the Clopper–Pearson exact method.

Supplementary Table S3. Multivariable logistic regression for advanced neoplasia among polyp patients aged 18–44.

Variable	Unadjusted OR (95% CI), P	Adjusted OR (95% CI), P
Age (per 1 year)	1.059 (1.019–1.102), 0.004	1.081 (1.036–1.128), <0.001
Sex (male vs female)	—	1.193 (0.782–1.820), 0.413
BMI (per 1 kg/m²)	—	1.098 (1.041–1.157), 0.001
Total cholesterol (per 1 mmol/L)	—	1.520 (1.130–2.045), 0.006
Triglycerides (per 1 mmol/L)	—	0.782 (0.603–1.015), 0.064
Fasting glucose (per 1 mmol/L)	—	0.421 (0.282–0.629), <0.001
Hypertension (yes)	—	0.940 (0.532–1.661), 0.832
Diabetes (yes)	—	0.761 (0.245–2.366), 0.637

AN = advanced neoplasia (high-risk adenoma or high-risk serrated polyp; CRC excluded per the primary definition). Complete-case analysis among 861/1021 polyp patients with complete data; 138 AN events. The unadjusted model includes age only; the adjusted model includes all listed variables. The unexpected inverse association of fasting glucose is likely a collinearity artifact and should not be over-interpreted.

Supplementary Table S4. Full-cohort multivariable logistic regression for adenoma and advanced neoplasia among first-detection colonoscopy examinees (asymptomatic screening + symptomatic), adjusting for age, sex, and colonoscopy indication.

Outcome (events / N)	Predictor	Unadjusted OR (95% CI), P	Adjusted OR (95% CI), P*
Adenoma (508 / 3218)	Age (per 1 year)	1.087 (1.067–1.108), <0.001	1.092 (1.071–1.113), <0.001
	Male sex (vs female)	—	1.862 (1.526–2.272), <0.001
	Symptomatic (vs asymptomatic)	—	1.295 (1.046–1.605), 0.018
Advanced neoplasia (134 / 3218)	Age (per 1 year)	1.088 (1.050–1.128), <0.001	1.091 (1.053–1.131), <0.001
	Male sex (vs female)	—	1.766 (1.230–2.536), 0.002
	Symptomatic (vs asymptomatic)	—	1.097 (0.752–1.601), 0.631

*OR, odds ratio; CI, confidence interval. Age entered as a continuous variable (per 1 year). *Adjusted for age, sex, and colonoscopy indication (symptomatic vs. asymptomatic health check-up). The analysis comprised first-detection examinees with a documented indication (n = 3218); surveillance colonoscopies for previously documented colorectal neoplasia and examinees without a documentable indication were excluded per protocol. Age remained independently associated with both adenoma and advanced neoplasia after adjustment for indication, with estimates essentially unchanged from the unadjusted values. BMI, metabolic, smoking, and comorbidity data remained unavailable for the broader cohort and could not be entered (see Limitations).*
